# Nanofabrication of Nonfouling Surfaces for Micropatterning of Cell and Microtissue

**DOI:** 10.3390/molecules15085525

**Published:** 2010-08-10

**Authors:** Hidenori Otsuka

**Affiliations:** Department of Applied Chemistry, Faculty of Science, Tokyo University of Science, 1-3 Kagurazaka, Shinjuku-ku, Tokyo 162-8601, Japan; E-Mail: h.otsuka@rs.kagu.tus.ac.jp; Tel.: +81-3-5228-8265; Fax: +81-3-5261-4631

**Keywords:** micropatterning, spheroid, co-culture, 3D culture, non-fouling surface, cell-cell interactions

## Abstract

Surface engineering techniques for cellular micropatterning are emerging as important tools to clarify the effects of the microenvironment on cellular behavior, as cells usually integrate and respond the microscale environment, such as chemical and mechanical properties of the surrounding fluid and extracellular matrix, soluble protein factors, small signal molecules, and contacts with neighboring cells. Furthermore, recent progress in cellular micropatterning has contributed to the development of cell-based biosensors for the functional characterization and detection of drugs, pathogens, toxicants, and odorants. In this regards, the ability to control shape and spreading of attached cells and cell-cell contacts through the form and dimension of the cell-adhesive patches with high precision is important. Commitment of stem cells to different specific lineages depends strongly on cell shape, implying that controlled microenvironments through engineered surfaces may not only be a valuable approach towards fundamental cell-biological studies, but also of great importance for the design of cell culture substrates for tissue engineering. To develop this kind of cellular microarray composed of a cell-resistant surface and cell attachment region, micropatterning a protein-repellent surface is important because cellular adhesion and proliferation are regulated by protein adsorption. The focus of this review is on the surface engineering aspects of biologically motivated micropatterning of two-dimensional surfaces with the aim to provide an introductory overview described in the literature. In particular, the importance of non-fouling surface chemistries is discussed.

## 1. Introduction

Surface engineering techniques for cellular micropatterning are emerging as important tools to clarify the effects of the microenvironment on cellular behabior [1,2,3,4,5,6,7,8,9], as cells usually integrate and respond to the microscale environment, such as chemical and mechanical properties of the surrounding fluid and extracellular matrix, soluble protein factors, small signal molecules, and contacts with neighboring cells [10,11,12,13,14,15,16,17,18,19,20,21]. Furthermore, living cells undergo physiological changes in response to exposure to drugs and environmental perturbations, such as toxins, pathogens, or other agents, and thus high-throughput technologies using whole cells have also been developed [22,23,24,25,26,27,28]. Recent progress in cell culture and microfabrication technologies has contributed to the development of cell based biosensors for the functional characterization and detection of such drugs, pathogens, toxicants, odorants, and other chemicals. Comprehensive reviews on self-assembled nanomaterials and bio-hybrids have also introduced these contributions [29,30]. In the fields of toxicology and drug testing, *in vivo* work has an advantage over *in vitro* work in that it takes into account the entire biological system in determining the time-dependent response to a chemical challenge. However, it is often not possible to do *in vivo* chemical toxicity studies with human subjects. Therefore, the development of a microscale cell culture analogue system, as an *in vitro* human surrogate, is another promising area using cell culture and microfabrication technologies.

To develop this kind of cellular microarray composed of a cell-resistant surface and cell attachment region, micropatterning a protein-repellent surface is important because cellular adhesion and proliferation are regulated by protein adsorption. The different engineering approaches aiming at a precise control of cell adhesion and spreading, through chemically and spatially designed surfaces, are the main focus of this review. In particular, the importance of non-fouling surface chemistries is discussed.

## 2. Cell Patterning Techniques

Microfabrication techniques are used to generate patterns of cells on surfaces. This cellular patterning is a necessary component for cell-based biosensors, cell culture analogues, tissue engineering, and fundamental studies of cell biology. We have developed the dry etching (or plasma etching) technique applied to achieve cell micropatterning. The photolithographic technique is also highly developed and has been widely used for patterning cells. However, this technique has some disadvantages for certain biological applications (especially biocompatibility). Recently, a set of alternative techniques, such as microcontact printing, microfluidic patterning using microchannels, and laminar flow patterning, have been developed for use in biological applications. In this paper, three representative methods – dry etching, photolithography, and microcontact printing **–** are reviewed.

## 3. The Basis of Cellular Patterning; Non-Fouling Surface Chemistries

**Figure 1:**
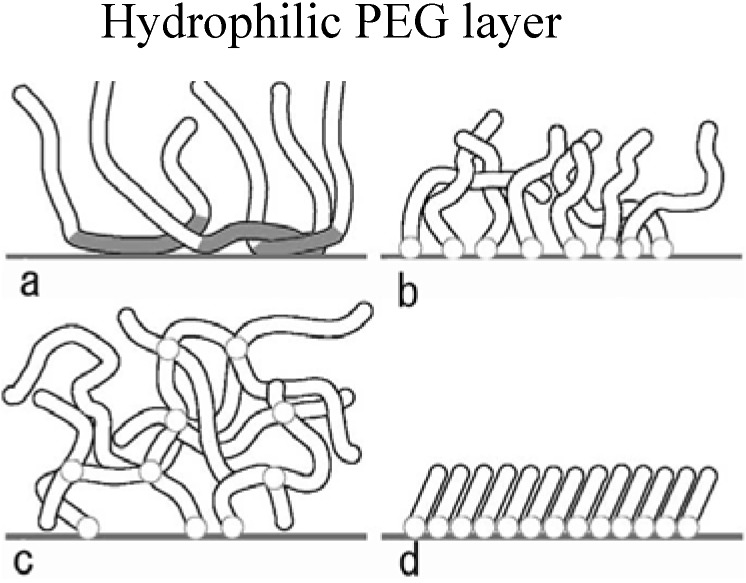
Schematics of the different methods for attaching PEG molecules to a surface: **(a)** adsorbed copolymers, **(b)** grafted macromolecules, **(c)** crosslinked thin-film hydrogels, and **(d)** self-assembled monolayers of short oligomers or macromolecules.

In any cellular patterning using surface modification, the ability to avoid non-specific interactions between the surface and the protein-containing media is crucial in order to generate unbiased experimental outcomes. Advances in surface chemistry have made possible the synthesis of so-called non-fouling surfaces that significantly reduce or eliminate the non-specific adsorption of proteins and other biomolecules from biological fluids such as cell culture media. Several types of native molecules have the ability to reduce the adsorption of proteins at surfaces, e.g., carbohydrates such as agarose and mannitol as well as albumin [31,32]. Due to their limited efficiency and stability, a number of synthetic materials have been developed [33]. The most widely used system is poly(ethylene glycol) or PEG with the monomeric repeating unit [–CH_2_–CH_2_–O–]– [also known as poly(ethylene oxide) or PEO]. The factors governing protein resistance of a PEG-graft co-polymer were recently investigated in detail, with quantitative information provided on the interfacial architecture of PEG chains and their influence on protein resistance [34,35,36,37,38,39,40,41,42,43,44,45,46,47,48,49,50,51]. Several theories have been proposed to explain PEG’s antifouling behavior, including its large excluded volume, osmotic repulsion, high molecular mobility, lack of protein binding sites, and high hydrophilicity. Protein-resistant PEGylated surfaces are often portrayed as near-liquid assemblies of highly mobile molecules and oligomeric segments that offer few binding sites to most proteins, as well as very short PEG-protein interaction times. Many different PEG surface-immobilization strategies have been successfully applied (Figure 1). The simplest and least stable PEGylated surface is made by the adsorption of PEG homopolymer directly onto a substrate. Such studies have been performed on various substrate materials, including glass and polymer. In all cases, the aount of adsorbed protein is reduced compared with unmodified surfaces. Because of its amphoteric nature, PEG adsorbs only weakly onto most surfaces and alternate strategies incorporate endgroups or copolymer blocks, which preferentially adsorb onto hydrophobic surfaces. A good example is the family of triblock copolymers of PEO-PPO-PEO [52,53,54,55,56,57,58], known as Pluronics. The hydrophobic PPO block - formed by substituting a methyl group for one of PEO’s hydrogens – adsorbs onto the surface, leaving the PEO segments to move freely in the aqueous solution [Figure 1(a)]. Surfaces coated with this copolymer show reduced adsorption of albumin, globulin, and fibrinogen, as well as reduced adhesion of platelets and macrophages. By changing the ratio of the PEO and PPO block lengths, the amphiphilic character of the block copolymer can be varied, which affects the hydrophobicity of the surfaces, as well as protein adsorption and cell adhesion [59,60,61,62,63].

We have reported novel approaches for the construction of functionalized poly(ethylene glycol) (PEG) layer on surfaces using heterobifunctional PEG/polylactide (PLA) block copolymers [64,65]. One of the objectives in the study is to investigate the effect of the variation in PEG chain length on surface properties. This includes assays on protein adsorption and cellular attachment to get a biochemical insight into the behavior of tethered PEG under biological conditions. For this purpose, numerous acetal-PEG/PLAs with different lengths of both PEG and PLA were synthesized. Molecular weights (MW) of PEG/PLA segments were abbreviated as follows: PEG/PLA(0.65/11.0, 1.8/7.0, 3.3/5.4, 5.0/4.6, 8.7/6.9) where the numbers in parenthesis denote the MW of the PEG segments and PLA segments in kg/mol, respectively. The PEG-brushed layer was constructed on the silanized glass surface by the spin coating of 4% (w/v) solution of PLA solution/toluene, followed by the 2% (w/v) solution of acetal-PEG/PLA/toluene. The wettability of the surface covered with PEG/PLA block copolymers was estimated both in air and in water by contact angle measurements (Table 1). In water-in-air measurements, coating of PEG/PLA block copolymer onto a PLA surface increased its wettability with increasing PEG molecular weight, as indicated by a decrease in static contact angle. A similar trend was observed in air-in-water measurements. The contact angle of a water droplet in air decreased remarkably in the range between PEG/PLA(0.65/1.10) and PEG/PLA(5.0/4.6). The decrease became moderate in the region with higher PEG molecular weights. Since the top few angstroms can be sensed by a contact angle measurement, the relatively high contact angles on the surfaces containing the lower molecular weight PEG is most likely to be attributed to an incomplete coverage of the uppermost surface by PEG chains. The dynamic contact angle was then measured to estimate the dynamics of the uppermost surface. The coating of PEG/PLA block copolymers reduces both the advancing and the receding angles of the substrates, although the change depends on the PEG molecular weight, which is consistent with the result of static contact angle. The maximum hysteresis was observed for the substrate with medium PEG chain length such as PEG/PLA(3.3/5.4). Hysteresis in the dynamic contact angle may be caused by the hydration of PEG segments. In the dry state, the PEG chain should assume a conformation flat to the surface experienced by the advancing contact line. Upon hydration, however, the PEG chain should extend from the surface due to the hydration of PEG chains. As a result, the receding contact line experiences a more hydrophilic surface than the advancing contact line. It is likely that this is the origin of the hysteresis observed on these surfaces.

**Table 1:** Molecular weights of PEG/PLA block copolymers and contact angle analysis.

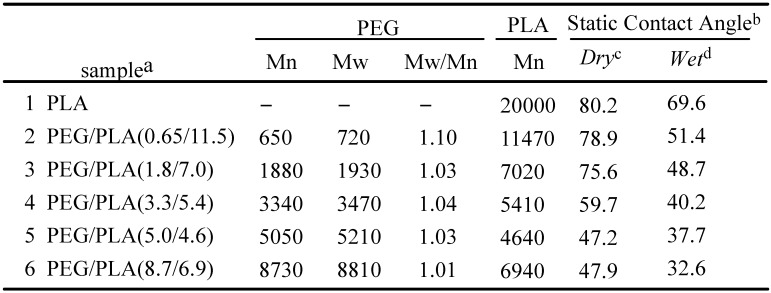

a) Molecular weights (MW) of PEG/PLA segments were abbreviated as follows: PEG/PLA(1.8/7.0, 5.0/4.6, 8.7/6.9) where the numbers in parenthesis denote the MW of the PEG segments and PLA segments in kg/mol, respectively; b) The values (degree) after hydration in PBS for 24h; c) The values in dry state were measured by a sessile droplet technique, where a water droplet was placed on the film surface; d) The values in wet state were measured by a captive bubble technique, where a sample film was immersed in water and a small air bubble was placed on the film from the bottom using a curved needle.

Protein adsorption on PEG/PLA surfaces was then estimated by using bovine serum albumin (BSA) as a model protein. On PLA surfaces BSA adsorbed significantly, while on PEG coated surfaces BSA adsorption is clearly decreased, mainly due to the steric stabilization by PEG chains. Moreover, minimum adsorption was obtained at a medium PEG chain length, *i.e.*, PEG/PLA(3.3/5.4); we note that this surface revealed the maximum hysteresis in dynamic contact angle measurement. Protein adsorption may be related to the hysteresis observed in the dynamic contact angle, which is likely to depend on particular surface properties such as the density and mobility of tethered PEG chains on the surface.

To enhance coating stability, many approaches have been studied to covalently bind PEG molecules to surfaces. These produce coatings that can vary from a monolayer to thin-film hydrogels [Figure 1(b,c)]. Two general approaches are used. One exploits the reactivity of the hydroxyl endgroup, which can be bound to surfaces such as activated silica. In order to increase the number of surface silanol groups, silica can be exposed to water plasmas. Silanols can then react with PEG hydroxyl groups to create an ester linkage to the activated surface. Surfaces modified in such a fashion are more hydrophilic, smoother, and demonstrate a reduced propensity for albumin adsorption. A second grafting approach replaces PEG’s hydroxyl endgroup with more reactive groups. The type of replacement group is determined by the target surface or the chemical reaction used in the process. Gombotz *et al.*, for example, used allylamine plasma glow discharge to introduce amine groups on the surface of poly(ethylene terephthalate), which were subsequently reacted with amine-terminated PEO using cyanuric chloride chemistry. A significant reduction in the adsorption of albumin and fibrinogen was achieved, despite an incomplete surface coverage. Similar results are found using silane chemistry to bind PEG molecules to the surface of Si and silica.

Another widely used PEG-chemistry-based approach relies on oligo-EG or PEG-modified alkanethiolate selfassembled monolayers (SAMs) [66,67,68,69,70,71,72,73,74,75,76,77,78] (Figure 1d). The thiol group couples to Au and other transition metals, which can be deposited as ultrathin films on substrates. Although these PEG-modified SAMs show substantial reduction of protein adsorption, they still adsorb significant amounts of serum proteins. Moreover, they tend to oxidize under ambient conditions restricting their use to short-term cell culture studies [79]. Bearinger *et al.* proposed an attractive alternative for modification of gold surfaces [80] based on PPS-PEG diblock or PEG-PPS-PEG triblock copolymers with poly(propylene sulfide) (PPS) as the central block (that binds to gold surfaces) and PEG grafted chains. Other polymer architectures such as gels and polymeric SAMs have also been successfully utilized to link PEG chains to surfaces. For example, Healy and coworkers developed a gel-like interpenetrating polymeric network (IPN) of poly(acrylamide) and poly(ethylene glycol) [P(AAm-co-EG)] [81,82], while Toner *et al.* used a poly(ethylene glycol) diacrylate (PEG-DA) hydrogel [83].

The choice of protein/cell resistant chemistry is often dictated by the type of substrate material to be used. Some chemistries are highly versatile and can be applied to different surfaces, while others require specific substrates as for example in the case of the gold-thiol system. However, each approach has its specific strengths and weaknesses. Covalently bound molecules have higher binding strength than physisorbed adlayers; however, non-covalent immobilization offers many attractive ways to modify surfaces. It should be pointed out that cell-patterning investigations frequently use serum-free or serum-depleted cell culture media. Some groups completely exclude serum from the media while others initially plate the cells in serum-free conditions and later add adequate amounts of serum to keep cells alive. These measures are sometimes necessary in order to prevent the cells to attach to the background, which would result in poor cell pattern quality. We note that such protocols have been frequently used in publications reporting the use of EG_3_ or EG_6_-modified alkanethiols. This is not so surprising since it is known that such coatings are not highly resistant to the adsorption of proteins.

**Figure 2:**
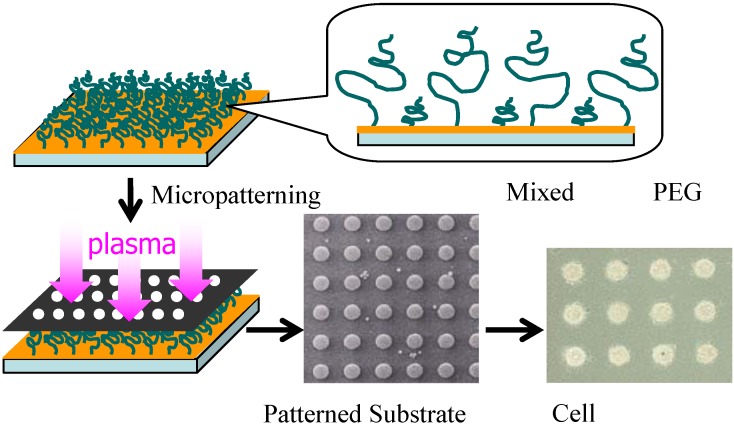
A two-dimensional microarray of endothelial cells was obtained on a micropatterned poly(ethylene glycol) (PEG)-brushed surface, based on the relationship between PEG chain density and cellular attachment.

Notably, our previous studies [84,85] have reported the long term stability of cell patterning (Figure 2). In the previous study, micropatterned PEGylated substrates with two-dimensional arrays of plasma-etched circular domains (*ϕ* = 100 μm) were prepared by sequential immobilization of PEG possessing a mercapto group at the end of the chain on the gold substrate, followed by plasma etching through a metal mask pattern with circular holes. 

**Figure 3:**
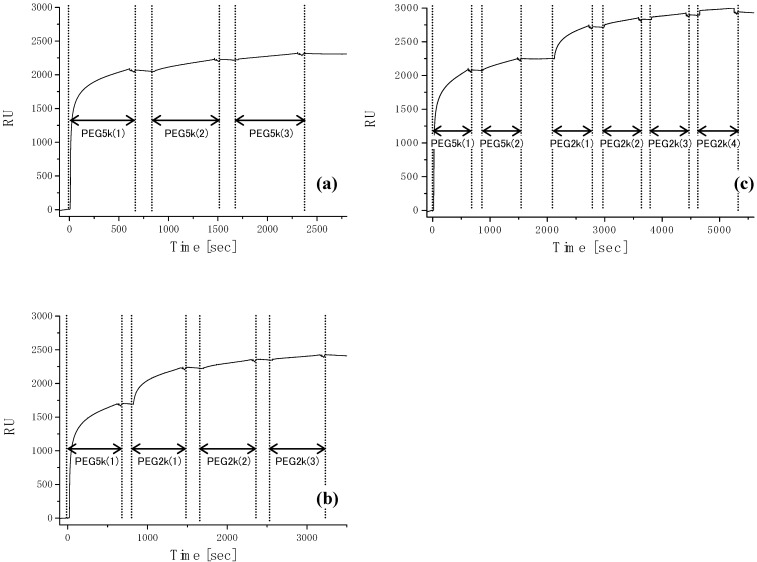
Sensorgrams of PEG immobilization on gold surfaces. **(a)** PEG5k(3), **(b)** PEG5k(1)/2k(3), and **(c)** PEG5k(2)/2k(4). Flow rate, 10 μL/min; running buffer, PBS (0.15 M, pH 7.4, containing 1 M NaCl); sample, 0.01 mg/mL of PEG (*M_w_*: 5k or 2k)/PBS (0.15 M, pH 7.4, containing 1 M NaCl) solution; sample injection, 100 μL for each time point [84].

The PEGylated region on the patterned substrate acts to repel proteins and thus inhibits cell adhesion. Proteins and cells are expected to adsorb from the serum-containing medium onto the plasma-etched circular domains, exposing the base gold surface. PEG chain density high enough to inhibit outgrowth of endothelial cells from the cell-adhering region in the horizontal direction could be obtained only by employing formation of a short filler layer of PEG (2 kDa, denoted as PEG2k) in the preconstructed longer PEG-brushed layer (5 kDa, denoted as PEG5k), which prevented nonspecific protein adsorption almost completely. Accordingly, the surface properties of the PEG coating were studied in detail to estimate protein adsorption and subsequent cell culture study on PEGylated surfaces. Three types of PEG immobilization [PEG5k(3), PEG5k(1)/2k(3), PEG5k(2)/2k(4)] were performed, as shown in SPR sensorgrams (Figure 3). After first treatment with PEG5k, the sensor surface was washed under running buffer to remove non-covalently adsorbed PEG. The sensor chip was then treated again with a solution of PEG5k. This cycle of adsorption/rinsing of PEG5k was repeated several times. Eventually, the total SPR angle shift was amplified by increasing the number of treatment cycles to three, indicating that repetitive treatment with PEG5k was effective in increasing the density of PEG [PEG5k(3)]. Notably, this trend became even more significant following additional treatment of the PEG5k surface with shorter PEG (PEG2k), as shown in Figure 3(b,c). We planned to increase the surface brush density by PEG2k, retaining the PEG5k brush surface character. Sensorgrams showed a number of interesting findings. First, immobilization of long-chain PEG [PEG5k(1)] increased markedly with changes in SPR angle [Figure 3(a-c)]. However, the extent of the shift decreased with the second injection of long-chain PEG [PEG5k(2)] [Figure 3(a,c)], and little change was seen on the third injection of long-chain PEG [PEG5k(3); Figure 3(a)]. On the other hand, immobilization of short-chain PEG [PEG2k(1)] after long-chain PEG resulted again in marked changes [Figure 3(b,c)]. These results suggested that long-chain PEG5k can hardly penetrate into the preconstructed longer PEG-brushed layer due to its exclusion volume effect, while short-chain PEG2k appreciably filled the gap in the preconstructed longer PEG layer. It should be noted that SPR sensorgrams showed a steep increase curve in PEG2k(1), as shown in Figure 3(b,c), indicating the importance of a short underbrushed PEG layer in increasing the PEG chain density.

**Figure 4:**
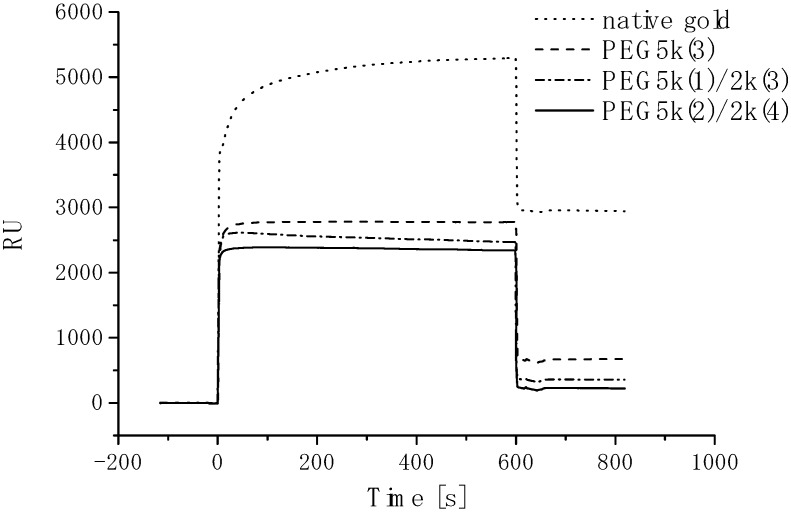
Sensorgrams of injection of serum containing cell culture medium (EBM-2 medium to culture HUVEC) on native gold and each PEGylated surfaces. Flow rate, 10 μL/min; running buffer, PBS (pH 7.4, 0.15 M); sample injection volume, 100 μL [84].

Nonspecific protein adsorption from the culture medium for HUVEC was estimated on each PEG-coated surface to estimate the cytophobicity of PEGylated surfaces, because the adsorbed proteins are responsible for subsequent cell adhesion. On bare gold as a control, the SPR angle shift due to the nonspecific adsorption of protein was 2927.4 RU, when serum-containing cell (HUVEC) culture medium (EBM-2) was passed over the surface. In contrast, PEG-coated surfaces clearly reduced protein adsorption (Figure 4). Figure 4 also shows a comparison of protein adsorption on the three types of PEG surface. The PEG5k(2)/2k(4) surface showed greatest degree of inhibition of protein adsorption from the medium, suggesting that the inhibitory effect of nonspecific protein adsorption was the highest for this surface among those studied. These results indicate that PEG surfaces with higher immobilized PEG chain density have greater ability to repel proteins. Based on these results, it was concluded that shorter PEG, *viz*. an underbrushed layer to increase the PEG surface density, played a substantial role in minimizing nonspecific protein adsorption. Other workers have also proposed that PEG mixtures which are polydisperse with respect to molecular weight are more efficacious than single molecular weights. Mixed PEGs were shown to have greatest efficacy in steric stabilization of colloidal particles and in protein repellency. The PEG5k(2)/2k(4) surface with the highest PEG chain density was expected to have the highest cytophobicity. In this way, a completely micropatterned array of endothelial cells with long-term viability was obtained. This clearly indicated the importance of a short underbrushed PEG layer in minimizing nonspecific protein adsorption for long-term maintenance of the active cell pattern.

## 4. Patterned 3D-Microorganized Cells Using Dry Etching (Plasma Etching)

*In vitro* culture of liver cells has received particular attention in biotechnology as many drugs fail in clinical studies either because they damage the liver directly or because liver metabolites are toxic. The study of hepatotoxicity would be greatly facilitated by the availability of *in vitro* culture systems that mimic real liver conditions. However, the development of liver-cell cultures as biosensors for drug toxicity faces challenges because of the difficulty in maintaining the differentiated phenotypes. In the liver, hepatocytes are found in a complex 3D environment in which nutrients, soluble factors and oxygens are transported through blood capillaries and bile canaliculi. Using silicon as a substrate, perfused 3D liver reactors have been fabricated on arrays of 300-μm-wide channels (capillaries) that comprise a scaffold for the ECM. Seeding hepatocytes with pre-aggregated multicellular spheroids in the 3D reactor generates cultures that are viable for a long time period (~3 weeks) and that exhibit a stable differentiated phenotype. Cells in 3D liver cultures also have cell-cell contacts, such as tight junctions and desmosomes that resemble those found in tissues *in vivo*. It has been also observed that co-culture of hepatocytes with other cell types, including liver epithelial cells and Kupffer cells, prolongs the survival of cultured hepatocytes and helps maintain liver-specific properties such as albumin secretion. Using a micropatterned 2D co-culture system, it has also been shown that liver-specific functions increase with heterotypic cell-cell interactions. Only hepatocytes close to the heterotypic interface maintain their differentiated phenotypes in longer-time culture. Relative to conventional co-culture, in which seeding densities of two cell types are varied on a planar surface, micropatterning techniques afford greatly improved control of homo- and heterotypic cell-cell interactions. The ability to culture cells such as liver cells *in vitro* and to demonstrate protein and gene expression levels similar to those found in tissue suggests that microfabricated cultures could have applications in toxicology and could also serve as model systems for *in vitro* analogues of organ tissue.

**Figure 5:**
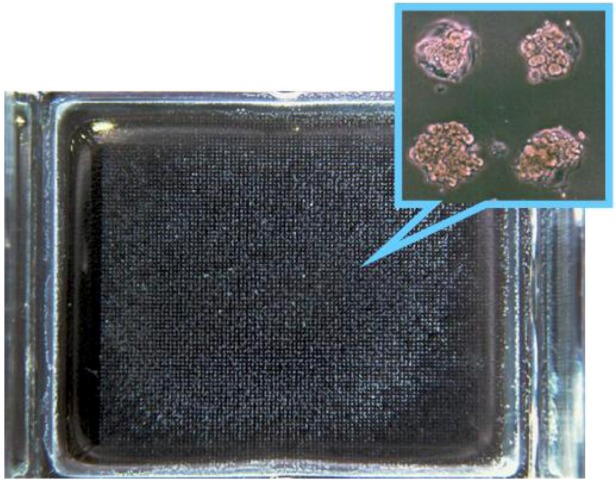
Micro-array of ten thousand (100 × 100) hepatocyte hetero-spheroids prepared on *ϕ*100-μm circular glass domains with *l*100 μm spacing on 20 × 20 mm glass substrate coated with α-lactosyl-PEG/PLA [28].

As shown in Figure 5, by using dry etching technique, we have constructed a two-dimensional microarray of ten thousand (100 × 100) hepatocyte hetero-spheroids, underlaid with endothelial cells, which was successfully constructed with a 100-μm spacing in an active area of 20 × 20 mm on micro-fabricated glass substrates that were coated with poly(ethylene glycol) (PEG) brushes [28]. Co-cultivation of hepatocytes with endothelial cells was essential to stabilize hepatocyte viability and liver-specific functions, allowing us to obtain hepatocyte spheroids with a diameter of 100 μm, functioning as a miniaturized liver to secret albumin for at least three weeks. Dry etching refers to the removal of material, typically a masked pattern of semiconductor material, by exposing the material to a bombardment of ions (usually a plasma of nitrogen, chlorine and boron trichloride) that dislodge portions of the material from the exposed surface. Unlike with many (but not all, see isotropic etching) of the wet chemical etchants used in wet etching, the dry etching process typically etches directionally or anisotropically.

**Figure 6:**
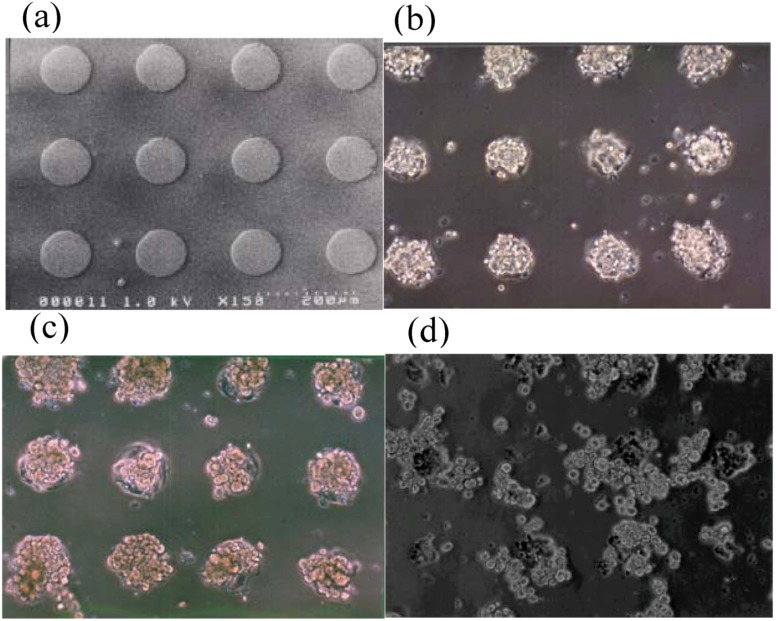
Patterned 3-D co-culture of hepatocyte spheroids and endothelial cells (BAECs). **(a)** Micro-patterned α-lactosyl-PEG/PLA coated dish with *ϕ*100 μm circular domains spaced in *l*100 μm intervals. **(b)** Patterned culture of BAECs on substrate (a) for 24 h at 37 °C. **(c)** Organized pattern of hepatocytes spheroids underlaid with BAECs. **(d)** Hepatocytes directly seeded on substrate (a) without pre-adhered BAECs [28].

In detail, micropatterned PEGylated substrates with two-dimensional arrays of plasma-etched circular domains (*ϕ*100-μm) were prepared by sequential spin-coating of polylactide (PLA) and α-lactosyl-poly(ethylene glycol) (PEG)/PLA block copolymer on silanized glass slide dishes, followed by plasma-etching through a metal mask pattern with circular holes [Figure 6(a)]. Round, 100-μm diameter holes separated by 100-μm (edge-to-edge distance) spacing was used to mask a N_2_+H_2_ plasma etch, forming the patterned α-lactosyl (or methoxy)-PEG/PLA surface. Bovine aortic endothelial cells (BAECs) at passage 13 were then seeded onto the patterned surfaces with *ϕ*100 μm circular domains that were edge-to-edge spaced in *l*100-μm intervals [Figure 6(a)], and cultured at 37 °C for 24 h in a 10% fetal bovine serum medium. Obviously, BAECs adhered only onto the circular domains exposing a glass substrate [Figure 6(b)]. Preferentially adsorbed extra-cellular matrix (ECM) proteins, including fibronectin, vitronectin, and laminin on the glass circular domains, may promote the adhesion of anchorage-dependent BAECs. Rat primary hepatocytes, suspended in a culture medium, were then applied to the patterned dishes with cultured endothelial cells selectively located in the circular domains. Interestingly, rat primary hepatocytes formed spheroids within 24 h only on the circular regions of existing endothelial cells, generating a 2D-arrayed structure of the hepatocyte spheroids [Figure 6(c)]. In contrast, on the same patterned α-lactosyl-PEG/PLA surface without pre-adhered BAECs, hepatocytes attached to and spread on both the PEG layer and the glass regions, without spheroid formation [Figure 6(d)]. 

**Figure 7:**
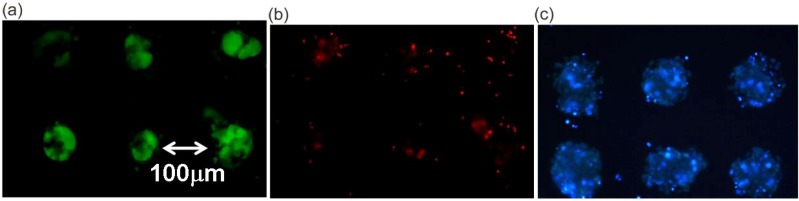
Viability assay by LIVE/DEAD Viability/Cytotoxicity assay kit of three dimensionally co-cultured spheroids on α-lactosyl-PEG/PLA-pattern-coated dishes for three weeks at 37 °C. **(a)** Live cell image stained with calcein. **(b)** Dead cell image stained with EthD-1. **(c)** Distinct nuclei stained with a DNA-binding dye (Hoechst 33342) [28].

These results demonstrate the significant role of BAEC as a feeder layer for the formation of hepatocyte spheroids. The cell viability of the obtained spheroid was assessed with a LIVE/DEAD Viability/Cytotoxicity assay kit. Living cells are distinguished by the presence of ubiquitous intracellular esterase activity, and are determined by the enzymatic conversion of the virtually nonfluorescent cell-permeant calcein AM to the intensely fluorescent and cell-impermeable calcein. The polyanionic calcein is well retained within living cells, producing an intense, uniformly green fluorescence (excitation/emission; ~494 nm/~517 nm). Indeed, the intense green fluorescence of calcein was observed intracellularly in the cytoplasm of every spheroid hepatocyte, even after three weeks of culture [Figure 7(a)]. It should be noted that no such green fluorescence was observed for isolated hepatocytes without any underlaid BAECs. On the other hand, ethidium homodimer-1 (EthD-1) enters only into cells with damaged plasma membranes and binds to nucleic acids, thereby producing a bright red fluorescence (excitation/emission; ~528 nm/~617 nm) in dead cells. No such red fluorescence was observed for spheroids underlaid with BAECs, in line with the result from calcein AM. In contrast, a bright red fluorescence was observed for isolated hepatocytes without underlaid BAECs [Figure 7(b)]. Staining with the fluorescent dye (Hoechst 33342) for nuclei further demonstrated nuclear morphology [Figure 7(c)]. These results suggest that cell viability was well retained in the spheroid-structure, interacting with the underlaid BAEC layer.

**Figure 8:**
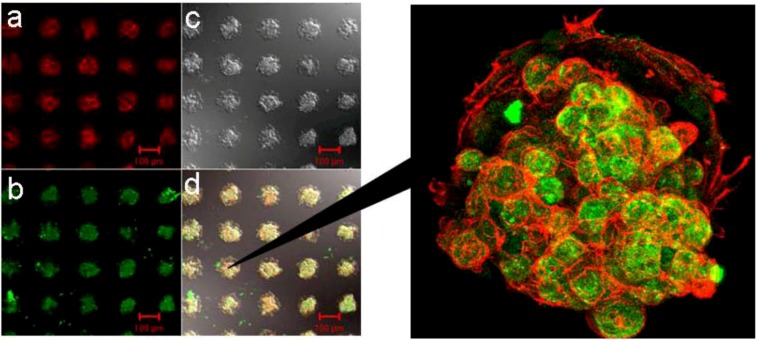
Confocal laser scanning microscopy of patterned three dimensional spheroids after the double staining of F-actin and albumin, co-cultured for three weeks at 37 °C (left). Spheroids were fixed and double stained with: **(a)** rhodamine-conjugated phalloidin for F-actin and **(b)** anti-rat albumin antibody and FITC-conjugated second antibody for albumin synthesis activity. **(c)** Interference reflection microscopy. **(d)** Superimposition of (a), (b), and (c). The four images were obtained from the same view field. Scale bars are 100 μm(right) 3-D view of spheroids, underlaid with endothelial cells as a feeder layer [28].

**Figure 9:**
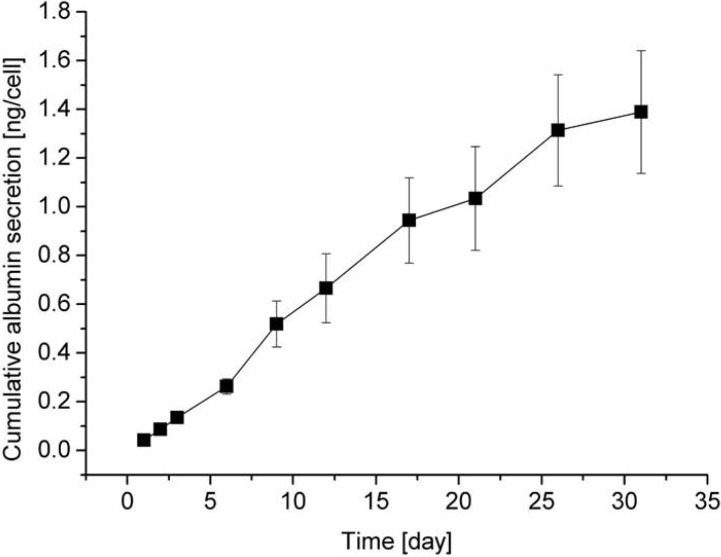
The change of albumin secretion from the hepatocyte hetero-spheroids underlaid with BAECs as a function of time [28].

Hepatocyte spheroids in contact with BAECs were characterized by an immuno-histochemical double staining method. In situ fluorescent staining was done with an anti-rat albumin antibody for cellular albumin synthesis, a characteristic phenotype of hepatocytes. Rhodamine-conjugated phalloidin was used for F-actin (Figure 8). This figure further demonstrates a 3-dimensional view of a multicellular spheroid of hepatocytes underlaid with BAECs as a feeder cell. This was reconstructed from a stack of 2-dimensional image volumes. It should be noted that spheroids significantly express a stable level of liver-specific functions (albumin secretion) even after three weeks, showing intense green fluorescence, compared to the usual cell monolayers. In multicellular organization intimately coupled to the dynamics of the actin cytoskelton, most of the actin was localized in the cell cortex, as opposed to the stress fiber which is linked to the cell-substratum contact via focal adhesion complex [86]. Obtained spheroids have ultrastructural similarities to the native liver tissue such as junctional complexes, leading to high level of retained liver-specific functions. Note that these albumin secretions, cytoskelton as well as cell-cell junction, are maintained intact in the spheroids, presumably due to the heterotypic cell interaction through the hepatocyte-BAEC contact [86,87,88,89]. To further investigate the cellular function in the hepatocyte hetero-spheroids, hepatic albumin secretion was evaluated as a function of time using a sandwich enzyme-linked immunosorbent assay (ELIZA). The results demonstrate that continuous albumin secretion in hepatocytes co-cultured with BAECs was observed for over 31 days of culture (Figure 9). Note that continuous secretion of albumin for 31 days has rarely been accomplished in other culture methods reported so far, and this is a direct demonstration that the surviving hepatocytes have functions comparable to the ones seen in the liver.

TBB and CBB (tissue and cell-based biosensors), based on the spheroid formation presented here, offer the promise of responding to environmental perturbations such as toxicants, pathogens or other agents in a physiologically relevant manner. In contrast to identification assay, such as those based on antibodies or nucleic acids that rely on structural determinants, cells respond only to biologically active threats. This miniaturized artificial liver array has the ability to rapidly evaluate drugs and environmental perturbations for potential risk to health, and to make predictions for effects of exposure. Furthermore, the patterned array of cell-organized structures such as spheroids on surfaces may have a particular importance for constructing tissue-engineered liver by seeding spheroids into three-dimensional scaffolds. This is also a useful tool to obtain insights into the mechanism of cell-cell interaction, a central research topic in cell biology.

## 5. Cell Assembly for Tissue Engineering

Artificial microtissues can also be fabricated by inducing the reaggregation of one or more cell types [91]. These microtissues may be beneficial for applications such as pancreatic, liver, vascular, and cardiac tissue engineering, as well as drug discovery. Layering of cells has been used to engineer myocardial tissues by assembling multiple sheets of cardiomyocytes [92] or to engineer blood vessels by fabricating cylindrically rolled sheets of endothelial cells [93]. Although such approaches may be suitable for some tissue engineering applications, they lack the complexity associated with the architecture of more complex organs. Microscale approaches may provide a solution to this challenge as templates to generate microtissues in a reproducible manner. For example, by using a combination of microcontact printing and micromachining, hepatocyte spheroids have been formed [94]. More recently, nonadhesive PEG microwells have been used as templates for formation of aggregates of various cell types, including ES cells [95]. This approach aims to overcome the disadvantages associated with the hanging drop and suspension culture methods [91] by providing control over the size, shape, and other features of the cellular assembly in a scalable manner. The controlled formation of embryoid bodies may also be important in generating more homogeneous cultures that are capable of directing the differentiation of ES cells. In addition, template-based assembly of cells could be used to organize multiple cell types into specific geometries relative to each other within these aggregated tissue sections. It is envisioned that with the integration of such technologies with biomaterials such as photocrosslinkable gels and microfluidics, more complex tissue sections for therapeutic applications can be fabricated. 

## 6. Photolithography

**Figure 10:**
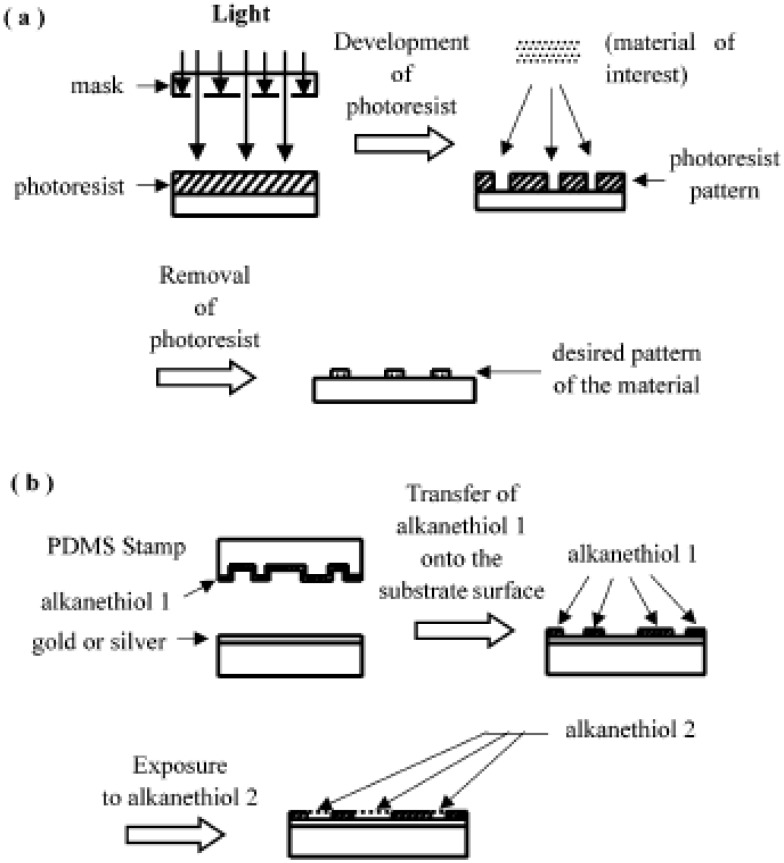
Schematics of the processes of micropatterning: **(a)** photolithography, **(b)** microcontact printing.

Photolithography on hard materials has been used widely for patterning cells [96,97,98,99,100,101]. In this technique, micropatterns are generated using light, photoresist, and mask, as shown in Figure 10(a). The photoresist is exposed to ultraviolet light through a mask containing the desired opaque pattern. The exposed part of the photoresist is then solubilized in a developer solution, resulting in a photoresist pattern. Subsequently, the materials of interest (e.g., cell-adhesion protein) are applied on the photoresist pattern, and the photoresist is then lifted off (e.g., by sonication in acetone). Examples of cell adhesion materials include polylysine, fibronectin, and collagen. Complex matrices such as Matrigel can also be used to achieve cell adhesion. Finally, the desired pattern of the material to which cells are specifically bound is obtained [98]. If the surface is incubated with cell solution, the desired cell pattern can be obtained. The photolithographic technique is highly developed for producing accurate patterns [102,104]; however, there are some disadvantages for the biological applications. Photolithography requires clean-room facilities and expensive equipment, and most chemicals used in this method are toxic to cells. Biological solutions are banned from clean room facilities originally designed for microelectronics applications, since ions and molecules cause harm to the finely tuned conductivity of a semiconductor circuit [104]. Surface modifications for introducing specific chemical functionalities or ligands are not easy in conventional photolithography [105]. Recently, Whitesides and colleagues have developed a set of alternative techniques, which are more suitable for biological applications [105,106,107]. They call it “soft lithography”, because a soft elastomeric material is used for pattern transfer or modification in those techniques.

The soft lithographic techniques mostly use poly-(dimethylsiloxane) (PDMS), since this material has appropriate properties. It is biocompatible, optically transparent, permeable to gases, elastomeric, and durable. Cells can be cultivated on the surface of PDMS, and the surface properties can also be readily modified.

## 7. Microcontact Printing

The microcontact printing method [108,109] is based on the pattern transfer of the material of interest from the PDMS stamp onto the substrate surface. The PDMS stamp is prepared by fabricating it from a master (typically microfabricated in silicon) having relief structures in photoresist on its surface (for details, see ref. [105]). Photolithography is used for the fabrication of masters in preparing PDMS stamps. Many of the studies involving the patterning of proteins and cells using microcontact printing have used selfassembled monolayers (SAMs) of alkanethiols on gold. As shown in Figure 10(b), the material of interest (e.g., an alkanethiol) is transferred from the PDMS stamp onto the substrate surface (e.g., gold or silver). The bare areas of substrate surface that the PDMS stamp has not touched can be exposed to another coating material (e.g., another kind of alkanethiol). The microcontact printing provides the patterning of self-assembled monolayers, and the resulting control over the adsorption of adhesive proteins facilitates the patterning of cells on substrates [108,109,110,111,112].

Chemicals to form SAMs typically have a chemical formula of Y(CH_2_)*n*X where Y is the anchor and X is the headgroup. The nature of the monolayer including its ability to self-assemble is influenced by *n*, or the number of methyl residues. The alkanethiols have a sulfide as the anchor group and a variety of headgroups. The binding of thiol to gold is very strong, resulting in a fairly stable surface. Typical headgroups are CH_3_ or COOH. The headgroup can greatly alter the hydrophobicity/hydrophilicity of the surface and protein and cell binding. The headgroup can be modified chemically. For example, an arginine-glycine-aspartate (RGD) peptide can be used as a headgroup that promotes cell attachment.

## 8. Conclusions

A number of techniques utilized for cell patterning have been separated in two categories: those where the cells are passively patterned by random seeding on surfaces modified with cytophilic and cytophobic regions, and those where the cells are actively deposited on the surfaces via optical or electrical forces or even directly printed. Although this paper has only reviewed the former category, we believe that there is a great potential for the future in combining specific techniques with the aim of solving a number of problems inherent to long-term culturing of active cell arrays.

Microfabrication using dry etching and photolithography is rapidly expanding, and “soft lithography” is being increasingly employed for biological applications [113]. Many microfluidic devices have also been developed, and these systems are rapidly being applied in the biomedical and pharmaceutical industries [114]. The fabrication of patterned surfaces and control of fluid flow will be useful in the wide range of fundamental studies of cell biology, including cell-surface adhesion, chemotaxis, cell-cell communication, and cellular ecology. The knowledge obtained from these studies will accelerate the development of cell-based biosensors and be applied to tissue engineering. Patterning technology promises to facilitate spatially controlled tissue engineering with applications in the regeneration of highly organized tissues.
